# Skeletal Muscle Metabolism: Origin or Prognostic Factor for Amyotrophic Lateral Sclerosis (ALS) Development?

**DOI:** 10.3390/cells10061449

**Published:** 2021-06-09

**Authors:** Cyril Quessada, Alexandra Bouscary, Frédérique René, Cristiana Valle, Alberto Ferri, Shyuan T. Ngo, Jean-Philippe Loeffler

**Affiliations:** 1 Mécanismes Centraux et Périphériques de la Neurodégénérescence, INSERM, UMR_S 1118, Centre de Recherche de Biomédecine de Strasbourg (CRBS), Université de Strasbourg, F-67000 Strasbourg, France; cyril.quessada@etu.unistra.fr (C.Q.); alexandra.bouscary@neuro-sys.com (A.B.); frederique.rene@unistra.fr (F.R.); 2 Neuro-Sys SAS, F-13120 Gardanne, France; 3 IRCCS Fondazione Santa Lucia, Institute of Translational Pharmacology, CNR, 00133 Roma, Italy; cristiana.valle@cnr.it (C.V.); alberto.ferri@cnr.it (A.F.); 4 Australian Institute for Bioengineering and Nanotechnology, The University of Queensland, Brisbane, QLD 4072, Australia; s.ngo@uq.edu.au; 5 Centre for Clinical Research, The University of Queensland, Herston, QLD 4006, Australia; 6 Queensland Brain Institute, The University of Queensland, St. Lucia, QLD 4072, Australia

**Keywords:** skeletal muscle, ALS, neuromuscular junction, hypermetabolism, PDK4, metabolic imbalance, trimetazidine

## Abstract

Amyotrophic lateral sclerosis (ALS) is a fatal neurodegenerative disease characterized by progressive and selective loss of motor neurons, amyotrophy and skeletal muscle paralysis usually leading to death due to respiratory failure. While generally considered an intrinsic motor neuron disease, data obtained in recent years, including our own, suggest that motor neuron protection is not sufficient to counter the disease. The dismantling of the neuromuscular junction is closely linked to chronic energy deficit found throughout the body. Metabolic (hypermetabolism and dyslipidemia) and mitochondrial alterations described in patients and murine models of ALS are associated with the development and progression of disease pathology and they appear long before motor neurons die. It is clear that these metabolic changes participate in the pathology of the disease. In this review, we summarize these changes seen throughout the course of the disease, and the subsequent impact of glucose–fatty acid oxidation imbalance on disease progression. We also highlight studies that show that correcting this loss of metabolic flexibility should now be considered a major goal for the treatment of ALS.

## 1. Introduction

In humans, the muscular system is divided into two distinct categories: smooth and striated muscles. The number, organization, and function of striated muscle require considerable energy consumption when compared to the entire human body. In this review, we first present a summary of amyotrophic lateral sclerosis (ALS). Second, we examine the role of muscle energy metabolism in the pathophysiology of ALS and we further discuss how targeting muscle offers an avenue for treating the disease.

### 1.1. Amyotrophic Lateral Sclerosis

**Figure 1:**
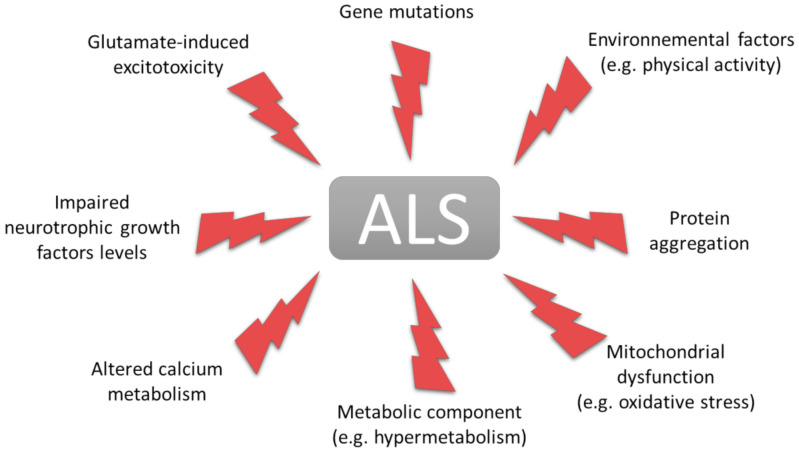
Pathogenic mechanisms thought to contribute to the development of ALS. Current data support several hypotheses that may explain the onset of ALS. Taken individually, these toxic events can mimic some of the hallmarks of ALS. This representation shows the complexity and multifactorial character of this disease.

Amyotrophic lateral sclerosis (ALS) is a fatal and incurable neurodegenerative disease. The gradual loss of weight and muscle strength and the onset and progression of muscle paralysis are the main visible presentations of this disease. From a cellular perspective, the loss of cortical motor neurons and spinal and bulbar motor neurons is clearly established [1]. ALS typically affects patients between the ages of 50 and 60 and invariably causes death 3 to 5 years after onset [2]. Worldwide, 60,000 people die of ALS each year [3] with an incidence of 1.75 to 3 per 100,000 per year [4]. In France, there are 3 to 4 new cases per year per 100,000 individuals and a prevalence of 6000 [5]. Many genes have been directly linked with the sporadic (90% of cases) and familial forms (10% of cases) of ALS [6]. In the latter, the pattern of inheritance is generally autosomal dominant. Among the main major susceptibility genes, *C9ORF72*, *SOD1*, *FUS* and *TARDBP* account for 48%, 12%, 5% and 5% in familial forms, respectively [5,6,7,8,9,10,11]. Currently, only two FDA-approved treatments are indicated for the treatment of ALS: Rilutek and Edaravone. These molecules increase the life expectancy of some patients by a few months [12,13]. The pathogenic mechanisms that are proposed to contribute to the degeneration of motor neurons include excitotoxicity, oxidative stress, protein aggregation, alteration of RNA metabolism and mitochondrial dysfunction [14,15,16,17,18,19,20] (Figure 1). Two hypotheses integrating these pathogenic mechanisms leading to ALS are currently proposed: the corticofugal dying-forward hypothesis which describes the progressive descending neurodegeneration, initiated in the motor cortex, spreading to the motor neuron and ultimately affecting the neuromuscular junctions (NMJ) [21,22,23] and the dying back hypothesis which is initiated at the NMJ with a retrograde progression of degeneration [24]. These two hypotheses are not mutually exclusive and may coexist to initiate ALS. The topic of this review is focused on the dying-back process of the motor neurons initiated at the NMJ and how skeletal muscle can be involved in ALS.

### 1.2. Neuromuscular Junction 

While ALS is clearly a complex disease, skeletal muscle is now being considered a key player in its pathogenesis. Although recent neurophysiological data obtained in ALS patients support an early hyperexcitability of cortical motor neurons, the dismantling of the NMJ is one of the first anatomical pathogenic events in ALS [25]. In both ALS patients and in SOD1 mouse models of ALS [26,27,28,29,30], the dismantling of the NMJ takes place before the degeneration of motor neurons [31,32,33,34], when no clinical motor signs are visible [35] (see Table 1). This supports the idea that motor neuron death is not the only cause of NMJ dismantlement but that degeneration begins in the most distal portion of the axon, namely the synapse. In addition, the safeguard of soma does not prevent the loss of NMJs [36,37]. Indeed, the destabilization of NMJs precedes motor neuron death and induces the dying-back phenomenon of motor neurons [24]. To clarify the reasons of this dismantling, several studies have been carried out. It was shown that the expression of mutant *SOD1^G93A^* and *SOD1^G37R^* specifically in muscle led to the loss of NMJ before the loss of motor neuron [38,39,40]. These data demonstrate that expression of SOD1 mutant limited to skeletal muscle is sufficient by itself to induce NMJ destabilization and may lead to motor neuron death. 

In the skeletal muscle of ALS patients [41,42] and in presymptomatic *Sod1^G86R^* mice, expression of Nogo-A (neurite outgrowth inhibitor) was significantly increased and was correlated with the severity of disease [43,44]. Nogo-A could therefore participate in the destabilization of the NMJ and therefore in the degeneration of axon terminals of motor neurons. Ablation of Nogo-A in the muscle of *Sod1^G86R^* mice was shown to prevent muscle atrophy and denervation and to prolong survival by 10%. Conversely, muscle overexpression of Nogo-A induced muscle atrophy and denervation, and significantly reduced the size of the NMJ [45]. Thus, these data suggest that Nogo-A plays a role in maintaining the integrity and stabilization of neuromuscular synapses in *SOD1^G93A^* mice. However, none of these processes would, by itself, be the sole cause of ALS but they could all contribute together to induce ALS.

## 2. Mitochondrial Failure and Oxidative Stress in ALS

### 2.1. Muscle Mitochondria and Respiratory Complexes 

One of the main features of ALS is mitochondrial dysfunction [19,46,47,48,49,50] (Figure 1). Defects in mitochondrial structure [51,52,53] and function have been observed in the skeletal muscle of sporadic ALS patients [54,55] and animal models of ALS [56,57] (Table 2). 

Moreover, early abnormalities in mitochondrial dynamics contribute to the degeneration of motor neurons in culture [61] and may contribute to the pathophysiology of ALS [56]. In addition, mitochondrial calcium overload occurs in the nerve endings of ALS patients [29] and disruption in calcium homeostasis was seen in different cell models expressing mutant SOD1 [62,63], and in the CNS [62,64] and the skeletal muscles of *SOD1^G93A^* mice [57]. Moreover, in asymptomatic ALS mice, mitochondria of muscle cells are no longer able to regulate calcium signaling around NMJs, and an excessively high concentration of calcium in the cytosol may contribute to the progression of muscle atrophy in ALS [57,65]. These data confirm a close link between mitochondrial dysfunction and calcium deregulation, where the latter would consequently cause a defect in the mitochondrial respiratory chain, triggering a vicious cycle. 

The main mitochondrial abnormalities found in ALS concern respiratory complexes. Wiedemann and colleagues [58] reported severe deregulation of the respiratory chain complex I, and a decrease in the activity of respiratory complexes I and IV in the muscle of sporadic ALS patients as well as in the muscles of the *SOD1^G93A^* mouse model from a presymptomatic stage [51,54,55,59,66,67]. Mitochondrial functions also become progressively impaired when disease progresses [55] and abnormalities in mitochondrial DNA result in decreased activity of certain enzymes (e.g., NADH, COX) [59]. In addition, a significant induction of UCP3 protein has been observed in the muscles of ALS patients and ALS mice [68]. UCP3 is an uncoupling protein mainly expressed in the mitochondria of skeletal muscles, and overexpression of UCP3 in this tissue would induce an increase in lipid oxidation (β-oxidation) and energy expenditure [69,70]. Finally, the overexpression of UCP1 in *Sod1^G86R^* mice leads to the degeneration of motor neurons, dismantling of the NMJ and decreased survival [71]. Importantly, the alterations observed in skeletal muscle can be detrimental to the integrity of the NMJ [39] without necessarily representing a causal link [72]. Despite data indicating a strong relationship between mitochondrial abnormalities and the progression of ALS, it is still impossible to establish a causal link between these two phenomena [39,71,72,73,74].

### 2.2. ROS and Oxidative Stress 

Reactive oxygen species (ROS) are very short-lived metabolites produced during oxidative phosphorylation. Under normal physiological conditions, a cell consumes oxygen to produce energy, and at the same time must eliminate the ROS produced via defense mechanisms such as superoxide dismutase (SOD) and antioxidant metalloenzymes [75]. Under conditions of oxidative stress and reduced mitochondrial respiration, large amounts of ROS are produced and lead to cellular damage such as inflammatory response, excitotoxicity, protein aggregation and apoptosis [76,77]. Furthermore, increased β-oxidation of fatty acids leads to the generation of lipid by-products which contribute to lipotoxicity and to ROS production [78,79]. Several studies have already demonstrated the implication of oxidative stress in aging and in ALS [14,80,81]. Abnormally high levels of ROS markers were observed in fluids [82,83] and post-mortem tissues from sporadic ALS patients [84,85,86] (Figure 1). In muscle of *Sod1^G86R^* mice, oxidative stress was observed even before the onset of motor symptoms and obvious signs of denervation [87]. Moreover, in muscle of *SOD1^G93A^*, SOD1 activity was increased throughout ALS progression, indicating the presence of oxidative stress in muscle [81,88]. Dobrowolny and colleagues demonstrated that muscle expression of mutant *SOD1^G93A^* was sufficient to induce oxidative damage, muscle atrophy and dismantlement of the NMJs [38,73]. Recently, an increased production of ROS was shown in the muscle of *SOD1^G93A^* mice and in muscle of wild-type mice with transient overexpression of the *SOD1^G93A^* mutation [89]. Changes in mitochondrial functions were dependent on the progression of pathology, and the *SOD1^G93A^* mutation was found to directly contribute to mitochondrial dysfunction long before the death of motor neurons. Although the induction of oxidative stress was not sufficient to cause motor neuron death, the above evidence supports the contribution of uncontrolled ROS production in skeletal muscle to ALS development. However, other studies suggest that mitochondrial disorders in ALS are minor [66,90,91,92] but are increased as ALS escalates [55]. Altogether, these data support the hypothesis of a major involvement of oxidative stress and mitochondrial alterations in ALS progression. 

## 3. Metabolic Alterations in Amyotrophic Lateral Sclerosis

### 3.1. Discovery of Hypermetabolism 

Classically presented as a strict disease of the cortical, bulbar and spinal motor neurons, the alterations of skeletal muscle observed in ALS are often considered to reflect the loss of these neurons. However, many studies examining the involvement of altered energy metabolism in ALS are starting to challenge this dogma. One of the major symptoms of ALS is weight loss, which is often studied through the measurement of the body mass index (BMI). As ALS progresses, a reduction in BMI and body fat is reported in patients with ALS [93]. Early insulin resistance [94] and glucose intolerance [95] have also been reported in ALS patients. This insulin resistance, which leads to a decreased sensitivity of the peripheral tissues to insulin and limits nutrients entry into the cells, could participate to the reduced BMI seen in patients. The loss of BMI in ALS is also associated with malnutrition due to dysphagia, and a worse survival outcome [96,97], whereas a high BMI is linked to a lower risk of developing ALS [98,99]. BMI is therefore a prognostic factor for ALS [96,100,101,102,103].

**Figure 2:**
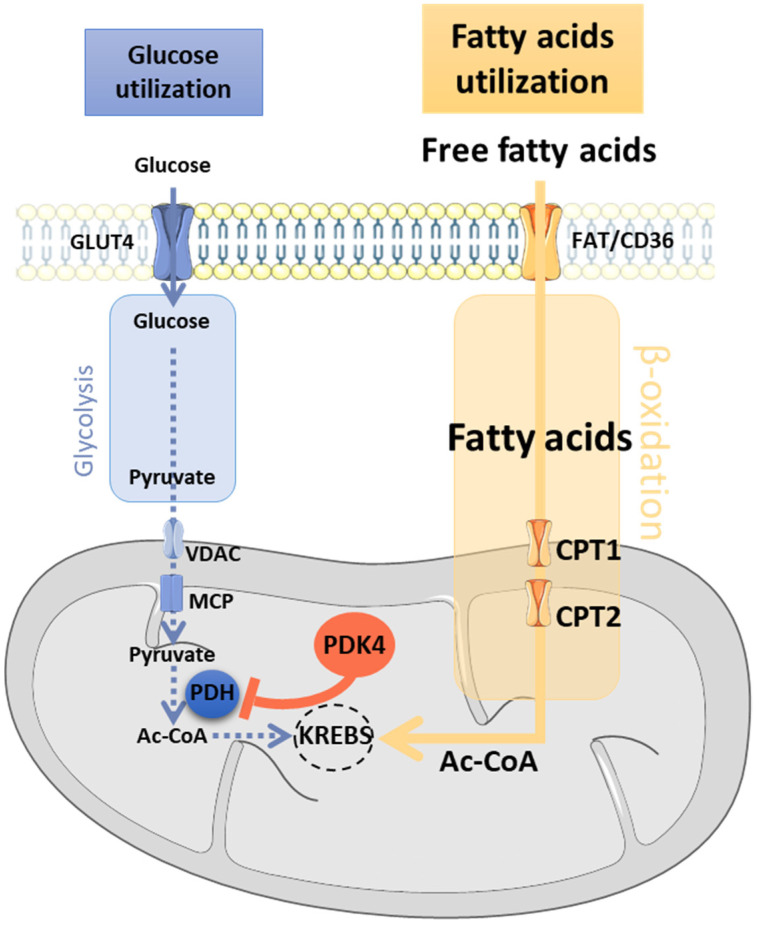
Altered energy metabolism in ALS skeletal muscle. In skeletal muscle, glucose enters into the cell through the GLUT4 transporter. It is then either stored as glycogen (not shown) or phosphorylated to give rise to pyruvate, the final product of glycolysis. Once formed, pyruvate enters into the mitochondria through a series of specific carriers (outer membrane: VDAC, inner membrane: MPC). In the mitochondria, pyruvate is oxidized into acetyl-CoA by PDH before entering the Krebs cycle to produce energy. Fatty acids enter the cell via FAT/CD36 transporters and get into the mitochondrial matrix thanks to CPT1 where β-oxidation will begin and produce acetyl-CoA which produces energy once in the Krebs cycle. In ALS, the oxidative pathway is greatly enhanced while the glycolytic pathway is reduced by abnormal induction of PDK4 in skeletal muscle which inactivates PDH by phosphorylation. PDK4 is believed to be at the root of this energy imbalance. Ac-CoA: acetyl-coenzyme A; CPT1: carnitine palmitoyltransferase 1; FAT/CD36: fatty acid translocase/cluster of differentiation 36; GLUT4: Glucose transporter type 4; MPC: mitochondria pyruvate carrier, PDH: pyruvate deshydrogenase; PDK4: pyruvate deshydrogenase kinase 4; VDAC: voltage-gated anionic channel.

In 2001, Desport et al. [104] identified an abnormal increase by 10% in resting energy expenditure in patients with ALS (*n* = 62; sex ratio M/F = 1.07) compared to healthy control group (*n* = 31). Defined as hypermetabolism, this phenomenon was subsequently confirmed, in a larger study, and was shown to be significantly increased by 14% and affected 62.3% of the 168 ALS patients (sex ratio M/F = 0.97) [105]. Data from the literature report that hypermetabolism affects up to 66% of ALS patients [104,105,106,107,108,109] and is an early event that persists throughout the course of disease [108,110,111]. Weight loss [112], hypermetabolism, and dyslipidemia are now considered as three major risk factors for ALS [107,109,113,114], and are associated with the severity of disease [108] (Figure 1). Remarkably, similar to what was seen in ALS patients, *Sod1^G86R^* and *SOD1^G93A^* mice were hypermetabolic and this metabolic change was already detectable at the clinically asymptomatic stage of the disease [115]. Moreover, an experimental induction of muscle hypermetabolism was sufficient to cause muscle denervation and motor neurons loss [71]. As such, it appears that hypermetabolism negatively impacts the progression of the disease. ALS mice also develop a loss of metabolic flexibility before any motor symptoms. This results in the inability to use glucose for energy production, leading to a decreased glycolysis and an increased β-oxidation in skeletal muscle [116] (Figure 2). 

In addition, this loss of metabolic flexibility preceded hypermetabolism in *SOD1^G93A^* mice [60]. Interestingly, both *Sod1^G86R^* [116] and *SOD1^G93A^* [60] mice had marked glucose intolerance from an early stage of ALS, and glucose intolerance is, with dyslipidemia, one of the key features of metabolic dysregulations in ALS patients [117]. One of the major players in this energy imbalance is pyruvate dehydrogenase kinase 4 (PDK4), the major muscle isoform of pyruvate dehydrogenase kinase [118,119], which plays a crucial role in balancing glucose–fatty acid flux. A significant induction of this metabolic marker was found in the skeletal muscles of *Sod1^G86R^* and *SOD1^G93A^* mice but also in the muscle of ALS patients [116]. The main metabolic changes are summarized below in Table 3.

According to these observations, it can therefore be hypothesized that correcting the energy balance (glucose vs. fatty acid) in skeletal muscle would reduce or slow down the development and/or progression of ALS.

### 3.2. Impairment of Skeletal Muscle Metabolism by Physical Activity

The muscular system requires a considerable supply of energy to handle a variety of physical challenges. To do this, skeletal muscle adapts its energy needs continuously, according to its environment. The selection of fuel source is based on the interaction between the metabolism of glucose and that of fatty acids and is controlled by the Randle cycle [127]. During short and intense exercise (e.g., sprinting), the involvement of fast-type glycolytic fibers necessitates that glucose oxidation (glycolysis) is favored as the primary fuel pathway. Conversely, during sustained moderate exercise (e.g., a marathon, jogging), which mobilizes slow type oxidative fibers, the oxidation of fatty acids (β-oxidation) is preferred [128]. Subjected to different conditions (e.g., intensive and repeated sports activity), muscle fibers adapt and change their phenotypic profile. For example, endurance training is correlated with a strong release of fatty acids and an improved fatty acid muscle absorption [129], thus reflecting improved β-oxidation and changes in muscle fiber type composition [130]. Intense physical exercise is increasingly studied in ALS due to the large number of patients diagnosed with ALS having had a sustained athletic career (Figure 1). Several studies reported a high risk of ALS for athletes such as soccer players, baseballers or tennis players [131,132,133,134]. However, one cannot exclude that other associated factors such as exposure to pesticides, doping agents or repeated injuries could be the cause or could participate in the development of the disease [135,136,137].

However, this link between high level athletes [133] or people with an intense lifestyle [138,139] and ALS is still debated. Indeed, some studies do not observe any link between physical activity and ALS [140,141] nor report activity as being a risk factor for the disease [142] given that ALS patients who perform moderate exercise in the clinic have improved ALSFRS scores [143]. As such, considering physical activity as a risk factor is highly controversial. Based on published data, it appears that if physical activity is not directly causative of ALS, it might in some cases worsen disease progression. Interestingly, in ALS mice, the type of physical exercise performed appeared to be either deleterious or protective. Indeed, Mahoney and his colleagues showed that high intensity exercise was detrimental to motor performance and survival in male *SOD1^G93A^* mice [144]. In contrast, in the same ALS mouse line, high frequency and large amplitude exercise, such as swimming, improved motor functions, delayed the loss of motor neurons, and significantly lengthened survival [125]. In addition, swimming had significant benefits on energy metabolism in the muscle, allowing it to reuse glucose as an energy source at the expense of lipids [120], while improving muscle strength [126]. Moreover, moderate exercise (low-speed treadmill running or free access to running wheel) significantly preserved motor performance as well as motor neuron density [145,146], unlike intense exercise (high-speed treadmill running) which slightly accelerated the onset of motor disorders [145]. The differences in effects between these two types of exercise result from the documented fact that swimming causes fast twitch fiber type transition and lactate production which promotes glucose metabolism [147]. Overall, the beneficial effects of swimming can be explained by the type of fibers recruited during this type of physical exercise. Swimming preferentially solicits fast fibers while endurance exercise recruits slow fibers [147]. These data demonstrate the benefit of preserving and/or stimulating glycolytic metabolism in skeletal muscle which is compromised in ALS.

### 3.3. The Metabolic Switch of Muscle Fiber Types in ALS

In ALS, fast-type synaptic connections are more vulnerable as the disease progresses, while slow-type synapses are relatively spared until the end stage of the disease [31]. In 2007, Hegedus and colleagues proposed that the loss of motor units occurred before the detection of key motor symptoms and loss of motor neurons [122]. In addition, in the *SOD1^G93A^* mouse model, they showed that contraction force of the tibialis anterior (TA), a glycolytic muscle, was reduced when compared to the gastrocnemius, an oxidative muscle. This observation was correlated with a selective and progressive degeneration of motor neurons innervating glycolytic fibers twitch (especially IIB fibers) [123]. It is now accepted that there is a change in muscle fiber types from glycolytic to oxidative in muscle of ALS patients [121], of *SOD1^G93A^* mice [123,124,125] and of mice expressing SOD1 mutant specifically in skeletal muscle [38,39,73]. Interestingly, at the onset of the disease, the entry of glucose into muscle fibers was not affected [115,148], suggesting that glucose is rerouted to glycogen stores, rather than being immediately used as a source of energy. Indeed, a decrease in glycogen synthase was observed at the presymptomatic stage of disease in *Sod1^G86R^* mice, and this was associated with the deterioration of glycolysis as well as unused glycogen stores in muscle [116]. In *SOD1^G93A^*, the proportion of glycolytic fibers was reduced when compared to oxidative fibers. This was consistent with the induction of oxidative myosin heavy chains and the repression of glycolytic myosin heavy chains [60,125]. Additionally, a muscle transition from a glycolytic to oxidative phenotype was described in *Sod1^G86R^* mice throughout the development of ALS [116]. In short, these studies demonstrate that metabolic changes in skeletal muscle are a hallmark of ALS, appear before the motor symptoms in mouse models and can have consequences at the NMJ. They further show that altered energy balance plays a role in the progression of ALS.

### 3.4. Main Actors of the Randle Cycle

To best ensure energy homeostasis, the Randle cycle continuously adapts specific fuel usage (glycolysis vs. ß-oxidation) to cellular demand by modifying blood glucose and free fatty acid concentrations via their respective GLUT4 and FAT/CD36 transporters (Figure 2). GLUT4 expression is drastically reduced in patients with ALS [149] and *SOD1^G93A^* mice [60,120] while FAT/CD36 is significantly increased in presymptomatic *Sod1^G86R^* mice [116]. Thus, the supply of glucose to muscle fibers is no longer assured, leading to insulin resistance [150,151,152] and glucose intolerance [94,95,153,154] in ALS. At the same time, the CPT1 transporter which allows the entry of fatty acids into the mitochondria was also overexpressed in the *SOD1^G93A^* [60] and *Sod1^G86R^* models [115,116]. These data are indicative of a disturbance in the assimilation of energy substrates and highlight metabolic imbalance at the level of the muscle. 

Once in the cell, glucose enters glycolysis or is stored as glycogen through the action of glycogen synthase. In *Sod1^G86R^* mice, at the asymptomatic stage, glycogen synthase activity and glycogen accumulation are significantly increased in skeletal muscle, suggesting that muscle cells are no longer able to use glycogen to produce energy, reflecting a problem with carbohydrate metabolism [116]. Regarding glycolysis, one of the first enzymes to be affected in ALS is phosphofructokinase 1 (PFK1). Indeed, from a presymptomatic stage, the expression of PFK1 and its activity were significantly reduced in the muscle of *Sod1^G86R^* mice [116]. The decrease in PFK1 expression in *Sod1^G86R^* mice could be a consequence of an increased uptake of fatty acid, enabled by induction of FAT/CD36 and CPT1 transporters, known to strongly inhibit PFK1 [155]. The reduction in PFK1 expression and activity occurs in response to an overexpression of PDK4 and high levels of PDK4 have been observed in the muscle of ALS patients, and in *SOD1^G93A^* and *Sod1^G86R^* mice even before any detectable sign of denervation [60,120]. While suggesting that the overexpression of PDK4 is not specific for mutations in the SOD1 gene, these data highlight that Randle cycle intermediates become altered in response to changes in glucose–fatty acid flux.

The expression of PDK4 depends on several transcription factors including Foxo1, PGC1α and PPARβ/δ. Foxo1 is an ubiquitous transcription factor that was strongly induced in muscles of *SOD1^G93A^* and *Sod1^G86R^* mice, as well as non-transgenic animals after sciatic nerve injury [116]. PGC1α plays an essential role in regulating the expression of genes involved in energy metabolism, lipid metabolism and in mitochondrial biogenesis [156,157,158,159,160]. Muscle overexpression of PGC1α leads to an induction of genes involved in the oxidative pathway, causing repression of glycolytic enzymes and glucose intolerance [161]. In mutant *SOD1^G37R^* mice, skeletal muscle induction of PGC1α led to maintenance of mitochondrial biogenesis, improved muscle function at the latter stages of disease [162], and was associated with an increase in oxidative type IIA fibers [163]. Alternatively, overexpression of PPARβ/δ in skeletal muscle increased the proportion of type 1 oxidative fibers [164], and constitutive overexpression of PPARβ/δ increases mitochondrial biogenesis and caused a switch from fast to slow fiber type [165]. 

Taken together, these studies demonstrate the crucial role of muscle energy balance in the pathogenesis of ALS, and are in agreement with the idea that correcting for, or preventing the loss of, metabolic flexibility could be a promising therapeutic approach in ALS.

## 4. Pharmacological Strategies for Targeting Energetic Imbalance in ALS

Below, we present a brief overview of two pharmacological modulators of energy balance that were studied for their possible repositioning as ALS treatments and a third one which is currently under investigation.

### 4.1. Dichloroacetate (DCA) 

**Figure 3:**
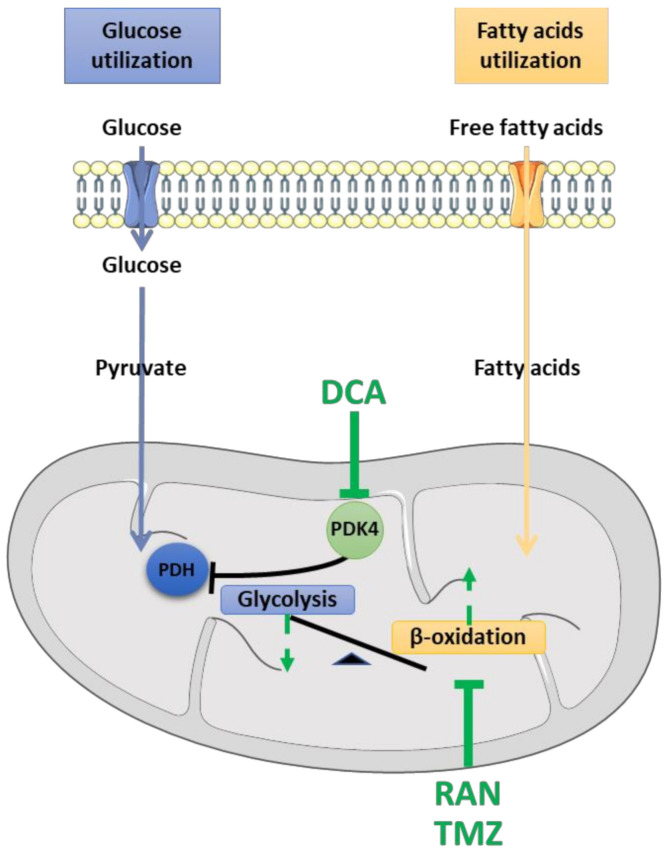
Metabolic reprogramming of skeletal muscle by pharmacological approaches.

DCA is a drug that restores optimal glucose oxidation while inhibiting the oxidation of fatty acids [166]. DCA inhibits the activity of PDK and thus stimulates the activity of PDH [167] (Figure 3). In 2012, Miquel and colleagues found that DCA improved mitochondrial function in astrocytes expressing the *SOD1^G93A^* mutation, while preventing their toxicity on cultured motor neurons. Additionally, they found that DCA improved muscle strength, preserved the integrity of NMJs, reduced motor neuron loss and prolonged survival in *SOD1^G93A^* mice [168]. In addition, DCA also delayed the onset of motor symptoms in *Sod1^G86R^* mice by limiting denervation and muscle atrophy [116]. The benefits observed in *Sod1^G86R^* mice could be explained by the protective effect of DCA on muscle fibers by restoring the energy balance or preventing its imbalance. Treatment with DCA limited the expression of genes controlling the oxidative pathway (e.g., PDK4, Foxo1 and PPARβ/δ) and increased the expression of those involved in the glycolytic pathway (e.g., PFK1). Moreover, DCA treatment improved motor functions, reduced the expression of denervation and atrophy markers, and limited oxidative stress [116]. These data therefore prove that restoring or preserving the metabolic balance can prevent oxidative stress, protect mitochondria, and prevent denervation and muscle atrophy in *Sod1^G86R^* mice. Although DCA exerts a number of beneficial effects, translation into the clinic is limited as long-term use of DCA leads to hepatotoxicity [169]. 

In ALS skeletal muscle, energy metabolism is disturbed: glycolysis is decreased while β-oxidation is increased. The three presented drugs could help normalize this energy imbalance. DCA can restore glycolysis by inhibiting PDK4 activity. RAN and TMZ inhibit β-oxidation. A correction of the glucose–fatty acid balance could explain the beneficial effects of these molecules found in various studies on ALS.

### 4.2. Ranolazine (RAN)

RAN is a β-oxidation inhibitor approved by the FDA for the treatment of angina pectoris [170,171,172,173]. RAN increases the oxidation of glucose [174,175,176] in patients with symptoms of chronic angina pectoris [177] or congenital myotonia [178] (Figure 3). Several studies have shown its efficacy against insulin resistance in animals [179] and patients with type 2 diabetes [180,181]. Despite its beneficial effects on energy metabolism, only one group of researchers working on ALS have exploited RAN efficacy. In 2020, RAN was shown to significantly improve motor functions, restore metabolic homeostasis of skeletal muscle, and prevent hypermetabolism in *SOD1^G93A^* mice. Due to the extreme severity of ALS, the positive effects of RAN could not be sustained until the final stage of the disease and could not affect the survival of animals [60]. Therefore, the putative utility of RAN remains an open question. Further preclinical studies, followed by clinical trials, are still needed at this stage to clarify whether RAN can be used as a clinically relevant drug to cure ALS.

### 4.3. Trimetazidine (TMZ)

TMZ is an anti-anginal and anti-ischemic agent [182,183] that inhibits the oxidation of fatty acids and promotes the oxidation of glucose [175,184,185]. The target of TMZ is the 3-ketoacyl thiolase, an enzyme involved in the last step of β-oxidation [184] (Figure 3). Recently, Ferraro and colleagues showed improved motor performance in a mouse model of sarcopenia after TMZ treatment [186]. In addition, this molecule increased the differentiation of C2C12 myoblasts and induced myogenesis in a tumor-bearing mouse model [187]. A recent study investigating the effects of TMZ on the peripheral nervous system demonstrated an antioxidant effect of TMZ, which resulted in a microenvironment conducive to nerve regeneration and increased remyelination [188]. Based on its pharmacological properties, TMZ appears as an interesting drug to test on ALS models. In preliminary experiments, TMZ significantly increased the motor functions of *Sod1*^G86R^ mice. We are currently pursuing these experiments to decipher the molecular mechanisms by which TMZ exerts its positive effects on ALS mice.

## 5. Open Questions and Future Directions

From recent works, it is now clear that ALS is not solely a neuronal disease but that the target of motor neurons, namely the skeletal muscle, is also a major player in disease initiation and progression. Further, the type of metabolism (e.g., glycolysis versus β-oxidation) is also decisive. There is clearly a shift in metabolism from glycolytic toward β-oxidation when disease progresses. This might account for the increased oxidative stress since, at a given ATP demand, producing the cellular fuel from β-oxidation consumes more oxygen and subsequently increases ROS production. We saw above that several molecules such as DCA, but also FDA-approved drugs such RAN or TMZ, can be repositioned for treating ALS to restore glycolysis in ALS mouse models and exert positive effects on muscle strength. Future studies should now be aimed at studying the effects of these drugs in ALS patients. 

## 6. Conclusions

In conclusion, ALS is a highly complex disease. ALS etiology and the multiple pathophysiological mechanisms that trigger the disease remain poorly understood. However, it is clear that skeletal muscle and its bioenergetic disturbances are involved in the development of the disease. Metabolic alterations observed in skeletal muscle in patients with ALS, and in mouse models of the disease prior to motor neuron degeneration, challenge the idea of ALS being a disease that originates from the neuron. Further investigations of muscle energy metabolism are essential and necessary to define new therapeutic approaches and to develop drug candidates for treating ALS.

## Figures and Tables

**Table 1:** Summary of the main NMJ alterations found in ALS patients and in ALS mouse models.

		Change Relative to Onset of Symptoms	Results	Ref.
**NMJ alterations**	Patients	Before	Decrease of motor unit number measured by MUNE in 2 asymptomatic SOD1 mutation carriers out of 19 (no statistics)	[30]
		After	33.8% of NMJ denervated in ALS (*n* = 10) vs. 9.8% in controls (*n* = 5) *p* < 0.05	[28]
	*SOD1^G93A^* mice	Before	P47: Denervation of 40% of NMJ in *medial gastrocnemius* (*n* = 890) (no statistics) P80: decrease by 60% of intact ventral root axons (*n* = 4/genotype, *n* < 0.01 vs. control)	[26]
			P58: Denervation of 78% of type IIb and 30% of type IIa fibers in *medial gastrocnemius* (2 mice/genotype; 20–30 muscle fibers analyzed; no statistics)	[31]
			P48: preferential denervation of type IIb NMJ followed by IIa and I in *gastrocnemius* (*n* = 3/genotype; 450 NMJ analyzed; no statistics)	[32]
			P30: 40% denervation of type IIb NMJ in *tibialis anterior* (*n* = 11/genotype; *p* ≤ 0.05)	[33]
			P30-36: Decrease by 15.5% of motor unit number measured by Baysian MUNE in *gastrocnemius* (*n* = 10/genotype; *p* = 0.018)	[34]

MUNE: motor unit number estimation.

**Table 2:** Summary of the main mitochondrial changes found in skeletal muscle of ALS patients and ALS mouse models.

		Change Relative to Onset of Symptoms	Results	Ref.
**Mitochondrial alterations in skeletal muscle**	Patients	After	Complex I activity reduced by 40% (*n* = 26 ALS, *n* = 28 controls; *p* < 0.01) Abnormal morphology (partially swollen, para-crystalline inclusions, vacuoles)	[51]
			Mitochondrial aggregates in the sub-sarcolemma zone in 49 out of 49ALS cases Ultrastructural abnormalities (giant mitochondria, para-crystalline inclusions) in 5 out of 49 ALS cases	[52]
			46% of patients with cytochrome c oxidase deficiency Respiratory chain complex activity decreased by at least 30% in patients with severe COX deficiency (8 out of 50)	[54]
			Increase of the maximal oxidative phosphorylation capacity of muscular mitochondria (*V*_max_) by 1.8 fold (*n* = 7 ALS, *n* = 7 controls; *p* < 0.05) Progressive decrease of complex IV activity as disease progresses (*n* = 7 ALS, *n* = 7 controls; *p* < 0.05)	[55]
			Complex I activity reduced by 47.5% (*n* = 14 ALS, *n* = 28 controls; *p* < 0.01)	[58]
			Complex I and IV activity reduced by 37.1% and 43.6% respectively (*n* = 17 ALS, *n* = 21 controls; *p* < 0.01)	[59]
	*SOD1^G93A^* mice	Before	P37: Localized loss of inner membrane potential near the NMJ Localized increase of calcium release by altered mitochondria after osmotic choc (*n* = 6/genotype; *p* < 0.0001)	[57]
			P55: Complex I activity reduced by 20% in the *tibialis anterior* (*n* = 5 independent experiments, *p* < 0.0001); P55: Decrease of oxygen consumption rate (OCR) by 30% (four independent experiments, with each sample tested in quadruplicate, *p* < 0.0001)	[60]

**Table 3:** Summary of the main alterations of metabolism and contractile properties of skeletal muscle in ALS patients and ALS mouse models.

		Change Relative to Onset of Symptoms	Results	Ref.
**Alteration of skeletal muscle metabolism and contractile properties**	Patients	After	3-fold increase of *PDK4* mRNA level compared to controls (ALS: *n* = 11, control *n* = 7; *p* = 0.035)	[116]
			4-fold increase of *PDK4* mRNA level and 20% decrease *GAPDH* mRNA level in ALS *vs* control cases (*anconeus* or *deltoid* muscle; ALS: *n* = 10, control: *n* = 6; *p* < 0.05)	[120]
			Switch in muscle fiber type from glycolytic to oxidative in muscle biopsies of ALS patients (ALS: *n* = 9, 200-300 fibers/biopsy, *p* < 001)	[121]
	*SOD1^G93A^* mice	Before	P55: *Pdk4* mRNA level is increased 2.5-fold in *tibialis anterior* (*p* < 0.05) P70: decrease of type IIb fiber mRNA level by 0.5-fold (*n* ≥ 4; *p* < 0.05) and *Glut4* mRNA level by 0.4-fold (*n* ≥ 4; *p* < 0.05), and increase of CPT1 by 4.8-fold (*n* = 4, *p* < 0.01) in *TA* →Bioenergetics defects	[60]
			P70: glucose tolerance is significantly decreased (*n* = 6/genotype, *p* < 0.05)	[120]
			P40: 22% decrease of motor unit number in the glycolytic gastrocnemius muscle compared to control mice (*p* < 0.05) P80: 45% decrease of tetanic force in the glycolytic muscle (extensor digitorum longus) and 48% decrease of motor units number compared to control mice (*p* < 0.05) → Sequential denervation of glycolytic muscles with disease progression	[122]
			P60: the tetanic contractile force developed by the glycolytic muscle *TA* is reduced by 80% (*p* < 0.01) and the number of motor units declines by 60% (*p* <0.01) compared to control mice P60: the number of innervated type IIb fibers is reduced by 40% in *TA* (*p* < 0.01) P60: decrease of cross sectioning area of type IIbfibers (*p* < 0.001) → Selective vulnerability of fast-twitch type IIb muscle fibers → Preferential denervation of fast motor neurons	[123]
			P60: decrease of fast-twitch muscle fibers diameter by 12.5% compared to control mice (*gastrocnemius* muscle, number of fibers analyzed: ALS *n* = 63, control *n* = 65; *p* < 0.01)	[124]
		After	Swimming straining started at P70 delays disease onset by 2 weeks (*p* < 0.001) and extends survival by 3 weeks (*p* < 0.01) (*n* = 8/genotype) P115: the fast-to-slow myofiber transition in the fast-twitch *plantaris* and *TA* are significantly limited by the swimming program	[125]
			P115: *Glut4* and *Gapdh* mRNA level are reduced by around 75% in the *TA* and *soleus* muscles (*p* < 0.05); Swimming training increases *Glut4* and *Gapdh* mRNA levels to control levels in TA but not in *soleus; Pdk4* mRNA level is increased by 2-fold in TA (*n* = 5/genotype, *p* < 0.05) → Impairment of glycolytic pathway → Physical activity improves metabolism	[120]
			P105: Swimming training started at P70 maintains grip strength in ALS mice (*n* = 8/genotype, *p* < 0.05 vs ALS sedentary mice P105: Citrate synthase activity is reduced by 30% in ALS sedentary mice compared to control (*p* = 0.0007) and swimming training prevents this decrease (*n* = 8/condition). P105: Malate dehydrogenase activity is increased ed by 25% (*n* = 8/condition, *p* < 0.0001) → Altered glucose metabolism → Swimming exercise modulates skeletal muscle energy metabolism	[126]
	*Sod1^G86R^ mice*	Before	P65: ALS mice have improved performance during endurance exercise P65: glucose handling is altered. In TA, glycogen stores are increased, PFK activity is decreased by 23% (ALS mice *n* = 7, control: *n* = 6; *p* = 0;016), pyruvate level is 1.7 fold increased (*n* = 5/genotype; *p* = 0;019) *Pdk4* mRNA level is 2.2-fold increased in TA compared to control mice (ALS mice *n* = 8, control: *n* = 7; *p* = 0;014) while unchanged in *soleus*. Relative mRNA levels of genes involved in lipid handling pathway (*Lpl; Cd36; Acsf2; Cpt1; PparB/∂*) are increased in TA →Metabolic switch: glycolytic pathway is strongly inhibited, and β-oxidation is enhanced	[116]
		After	P95: grip strength is decreased in ALS mice (*n* = 8/genotype, *p* = 0.03) and dichloraoacetate treatment prevents grip strength loss (ALS mice: *n* = 8; control: *n* = 9, *p* = 0.0003) DCA corrects the metabolic switch in TA	[116]

*Acsf2*: acyl-CoA synthetase family member 2; *Cd36/Fat*: fatty acid translocase; *Cpt1*: carnitine palmitoyltransferase 1; *Gapdh*: glyceraldehyde-3-phosphate dehydrogenase; *Glut4*: glucose transporter 4; *Lpl*: lipoprotein lipase; *Pdk4*: pyruvate dehydrogenase kinase 4; *Pfk*: phosphofructokinase; *Pparβ/δ*: Peroxisome proliferator-activated receptor β/δ; TA: *tibialis anterior.*

## Data Availability

Not applicable.
